# CD4 T-cell depletion prevents Lassa fever associated hearing loss in the mouse model

**DOI:** 10.1371/journal.ppat.1010557

**Published:** 2022-05-23

**Authors:** Junki Maruyama, Rachel A. Reyna, Megumi Kishimoto-Urata, Shinji Urata, John T. Manning, Nantian Harsell, Rebecca Cook, Cheng Huang, Janko Nikolich-Zugich, Tomoko Makishima, Slobodan Paessler

**Affiliations:** 1 Department of Pathology, The University of Texas Medical Branch, Galveston, Texas, United States of America; 2 Department of Otolaryngology, The University of Texas Medical Branch, Galveston, Texas, United States of America; 3 Department of Immunobiology and the University of Arizona Center on Aging, University of Arizona College of Medicine, Tucson, Arizona, United States of America; University of Pittsburgh, UNITED STATES

## Abstract

Lassa virus (LASV) is the causative agent of Lassa fever (LF), which presents as a lethal hemorrhagic disease in severe cases. LASV-induced hearing loss in survivors is a huge socioeconomic burden, however, the mechanism(s) leading to hearing loss is unknown. In this study, we evaluate in a mouse LF model the auditory function using auditory brainstem response (ABR) and distortion product otoacoustic emissions (DPOAE) to determine the mechanisms underlying LASV-induced hearing loss. In the process, we pioneered measures of ABR and DPOAE tests in rodents in biosafety level 4 (BSL-4) facilities. Our T cell depletion studies demonstrated that CD4 T-cells play an important role in LASV-induced hearing loss, while CD8 T-cells are critical for the pathogenicity in the acute phase of LASV infection. Results presented in this study may help to develop future countermeasures against acute disease and LASV-induced hearing loss.

## Author summary

Lassa virus (LASV) is known to cause lethal hemorrhagic fever, Lassa fever, and is classified into Risk Group-4 agents, which need to be handled in biosafety level-4 (BSL-4) due to their high pathogenicity. LASV is also known to cause hearing loss in patients. Hearing loss caused by LASV infection has a huge impact on patients’ quality of life and socioeconomics in endemic areas, however, its mechanism is unknown. In order to unveil the mechanisms underlying hearing loss in Lassa fever patients, we used auditory tests, which are also used in newborn screening, in a mouse model of Lassa fever and revealed that LASV causes hearing loss with the combination of virus replication and host immune response and that CD4 T-cells have an important role in LASV-induced hearing loss. This new finding gives us a new insight to develop vaccines against Lassa fever and also therapeutic methods to prevent hearing loss induced by LASV infection in the future.

## Introduction

Lassa virus (LASV) belongs to the family *Arenaviride* and is the causative agent of Lassa fever (LF). This zoonotic pathogen is maintained in its rodent reservoir, *Mastomys natalensis* [1], and human infections occur mostly through exposure to the aerosolized urine or feces of infected rodents or through direct contact with the blood, urine, feces, or other bodily secretions of a LF patient. LF onset begins with febrile, “flu-like” symptoms including fever, headache, sore throat, muscle pain, chest pain, nausea, vomiting, diarrhea, cough, and abdominal pain. In severe cases, LF develops into a hemorrhagic fever with facial edema, high fever, and bleeding from mucosal and gastrointestinal tracts [2]. The hospitalized case-fatality rate ranges from 15–70% depending on the outbreak [3–5]. An estimated 37.7 million people are at risk of contracting LASV, emphasizing the critical need for the development of safe and effective vaccines and therapeutics [6]. LASV is endemic in West African countries, such as Nigeria, Guinea, Liberia, and Sierra Leone, and outbreaks occur repeatedly [7]. Additionally, LASV causes sequelae in survivors that may be debilitating. Of note, approximately one-third of LF survivors develop sudden sensorineural hearing loss (SNHL), which is often bilateral and permanent [8–11]. Several viral infections, such as measles, mumps, rubella, and cytomegalovirus infection, are also known to cause SNHL in the acute phase of the disease [12], however, the LASV-induced SNHL mostly occurs during convalescent phase, sometimes weeks or months after leaving the hospital, and the exact mechanisms are largely unknown.

Stat1-knock-out (KO) mice are susceptible to lethal disease after LASV infection while immunocompetent inbred mice infected with LASV do not show any symptoms and efficiently clear the infection. LASV-infected Stat1-KO mice showed severe weight loss followed by hypothermia, and potential death [13]. Importantly, the majority of LASV-infected mice that survived the acute infection developed SNHL with high frequency [14,15], making this the only small animal model of reproducible LASV-induced SNHL. In our previous study, the acoustic startle reflex (ASR) was used to assess the auditory function in LASV-infected Stat1-KO mice. However, this is a behavioral test that is indirect and semi-quantitative, measuring the overall body movement induced by acoustic stimuli, which sometimes may be impacted by the severe infection alone. In order to overcome these problems, for the first time, we utilized two auditory function tests routinely used in human patients and newborns; a) auditory brainstem response (ABR) and b) distortion product otoacoustic emissions (DPOAE) [16]. ABR test evaluates the neural activity generated by sound stimuli along the auditory pathway from the auditory nerve to the brain, including the auditory brainstem and eighth cranial nerve [17]. ABR is useful for assessing neuronal function while otoacoustic emission (OAE) test is used to check the outer hair cell function as well as the overall health within the inner ear or cochlea. This test is used to measure otoacustic emissions, or OAEs, which are sounds given off by the sensory (hair cells) cells located in the inner ear when responding to sound [18]. These auditory tests are used for newborn hearing screening in humans and are also applicable to animals because of the objective, quantitative and non-invasive nature of these tests [19–21]. We have used both tests for the first time in the ABSL4 level facility, to achieve comprehensive analysis of the auditory function in LASV-infected mice.

Our previous study indicated that LASV-induced SNHL in Stat1-KO mice correlated with T-cell infiltration into the auditory ganglion [14]. To test the hypothesis that the T-cells play a role in LASV-induced SNHL, we performed a loss-of-function study by depleting T-cells through the injection of monoclonal antibodies against CD4 and/or CD8 T-cells. Here, we show the impact of T-cells on LASV infection-induced acute disease and the development of hearing loss in survivors by using quantitative and highly reliable auditory tests in the murine model of LF.

## Results

### LASV pathogenicity and LASV-induced hearing loss in Stat1-KO or wild type mice

**Fig 1:**
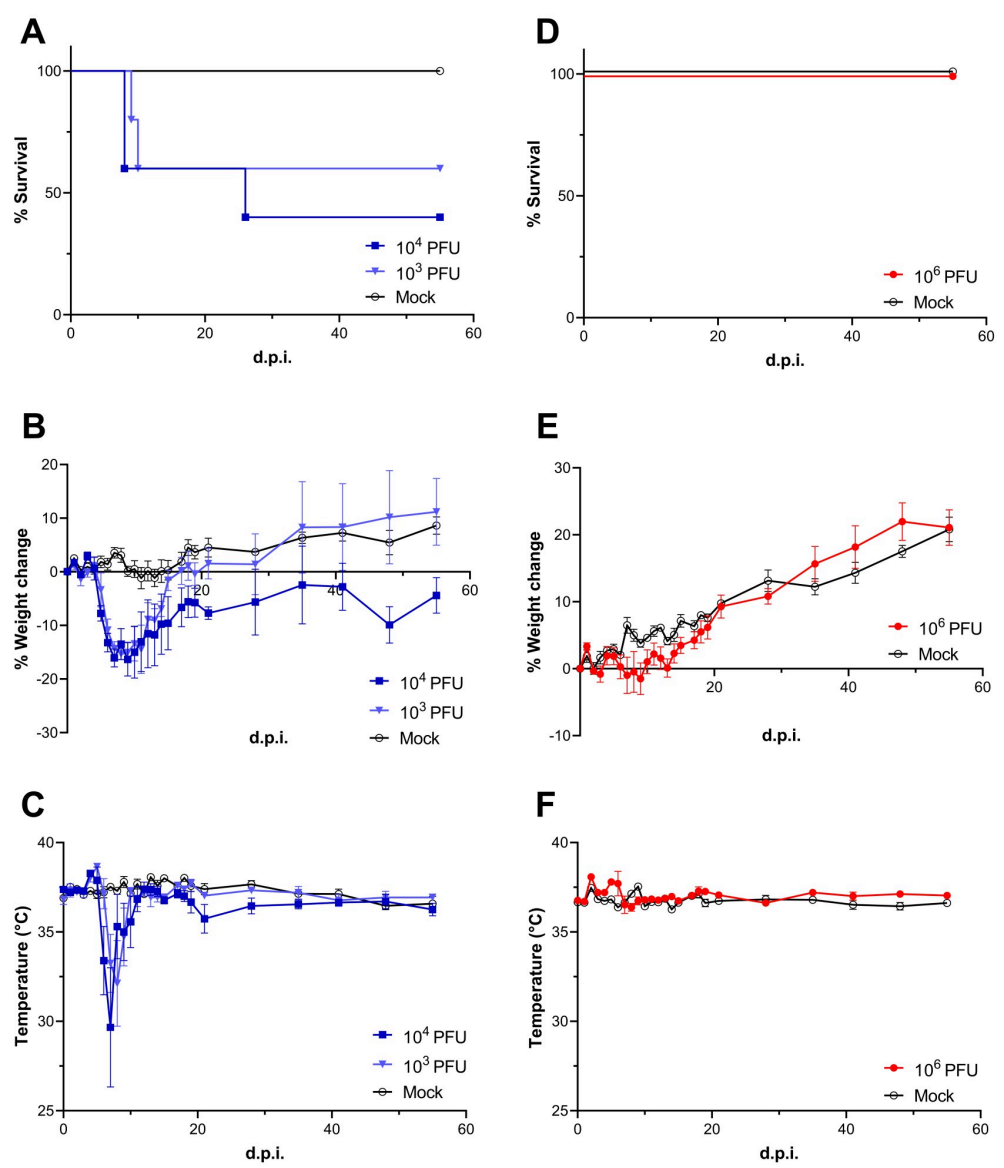
Pathogenicity of LASV in Stat1-KO and WT mice. Survival rate **(A and D)**, body weigh change **(B and E)**, and body temperature **(C and F)** of Stat1-KO **(A-C)** or WT mice **(D-F)** were plotted, respectively. Stat1-KO mice were inoculated 10^4^ or 10^3^ PFU of LASV (n = 10) and WT mice were inoculated 10^6^ PFU of LASV (n = 5). PBS was inoculated as a mock control into Stat1-KO (n = 10) or WT mice (n = 5). Means and standard error of mean (SEM) were shown **(B, C, E, and F)**.

129S6/SvEv-*Stat1*^*tm1Rds*^ (Stat1-KO) or wild type 129S6/SvEv (WT) mice were intraperitoneally infected with 10^3^−10^4^ plaque forming unit (PFU) or 10^6^ PFU of LASV respectively. PBS was injected into mice as a mock control. Similar to our previous report [13], Stat1-KO mice showed 60% survival with 10^3^ PFU LASV infection and 40% survival with 10^4^ PFU LASV infection, mostly succumbing between at 8–10 d.p.i. (Fig 1A). LASV-infected Stat1-KO mice started showing weight loss at 4 days post-infection (d.p.i.) and symptoms, such as scruffy coat, hunched back, and lethargy started at 6 d.p.i. simultaneously with hypothermia (Fig 1B and 1C). These symptoms disappeared at 12 d.p.i. in the surviving mice. One Stat1-KO mouse, which succumbed at 26 d.p.i., started showing neurological symptoms such as imbalance and body tremor at 22 d.p.i. On the other hand, WT mice did not show any symptoms except very mild weight loss between 7–9 d.p.i. and transient fever between 7–8 d.p.i. (Fig 1D–1F).

**Fig 2:**
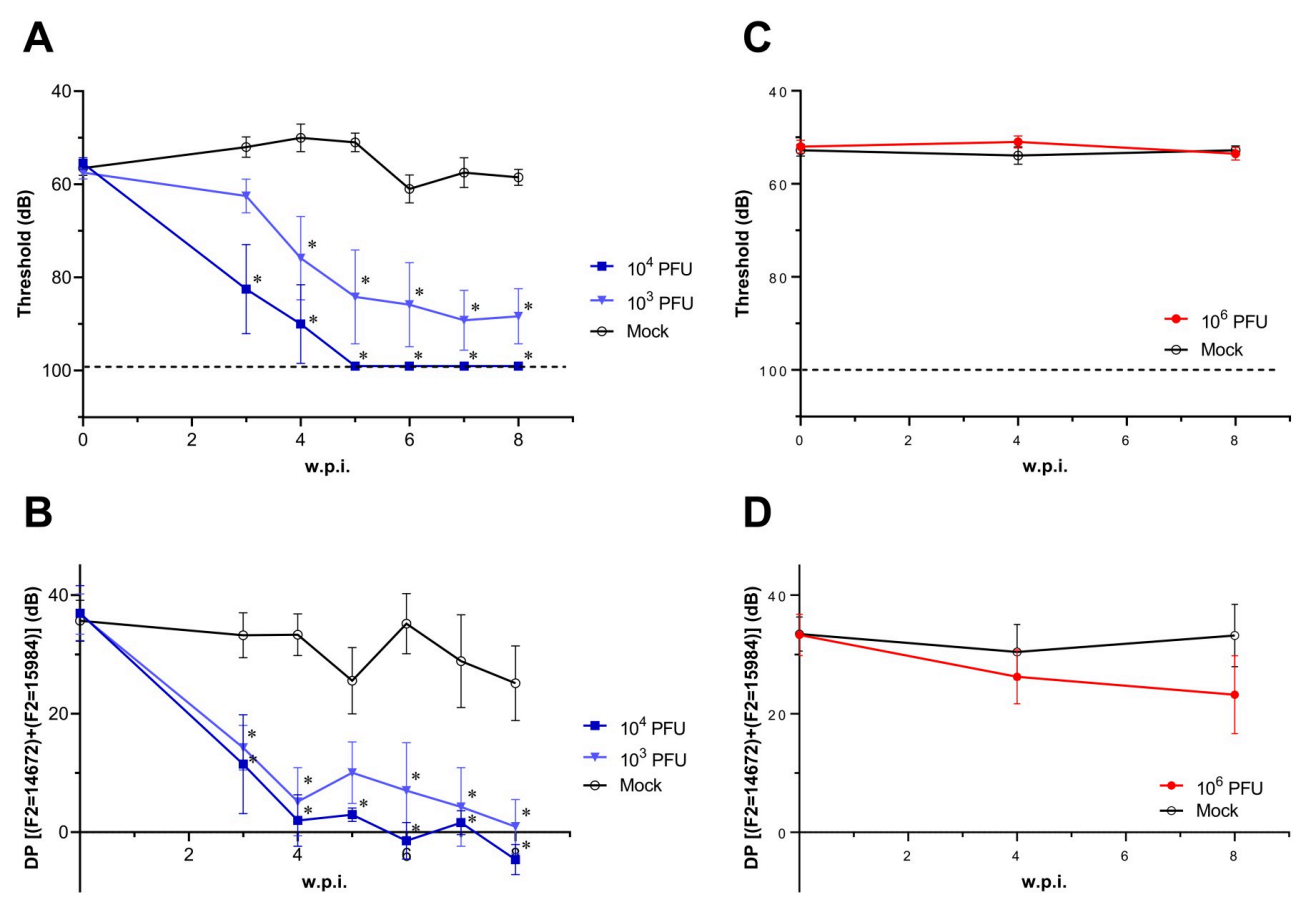
Hearing tests in LASV-infected Stat1-KO or WT mice. The results of ABR **(A and C)** or DPOAE **(B and D)** of LASV-infected Stat1-KO **(A and B)** (n = 6 for 10^3^ PFU; n = 4 for 10^4^ PFU) or WT mice **(C and D)** (n = 5) were plotted, respectively. Data were shown using the mean and SEM. The broken lines indicate the limit of detection in ABR (98< dB). Statistical differences at each time point compared to each mock group were calculated by 2-way ANOVA followed by Dunnett’s post hoc test, and shown in asterisks (p<0.05).

ABR and DPOAE was performed to assess the auditory function in the mice that survived LASV infection from 3 weeks post-infection (w.p.i.). LASV-infected Stat1-KO mice developed hearing loss, detected by both ABR and DPOAE, while LASV-infected WT mice did not develop any hearing dysfunction. (Fig 2 and S1–S4 Tables). In ABR, thresholds were significantly elevated around 3 or 4 w.p.i. in LASV-infected Stat1-KO mice as compared to the mock control group (Fig 2A and S1 Table). With DPOAE testing, significant decrease in DP values were observed around 3 w.p.i. in LASV-infected Stat1-KO mice as compared to the mock control group (Fig 2B and S2 Table). The mice that developped hearing loss once showed abnormal results in both of ABR and DPOAE through the end of the study, 8 w.p.i. On the other hand, WT mice did not show any significant differences in auditory function throughout the study (Fig 2C and 2D and S3 and S4 Tables).

### LASV pathogenicity and LASV-induced SNHL in T-cell-depleted Stat1-KO mice

**Fig 3:**
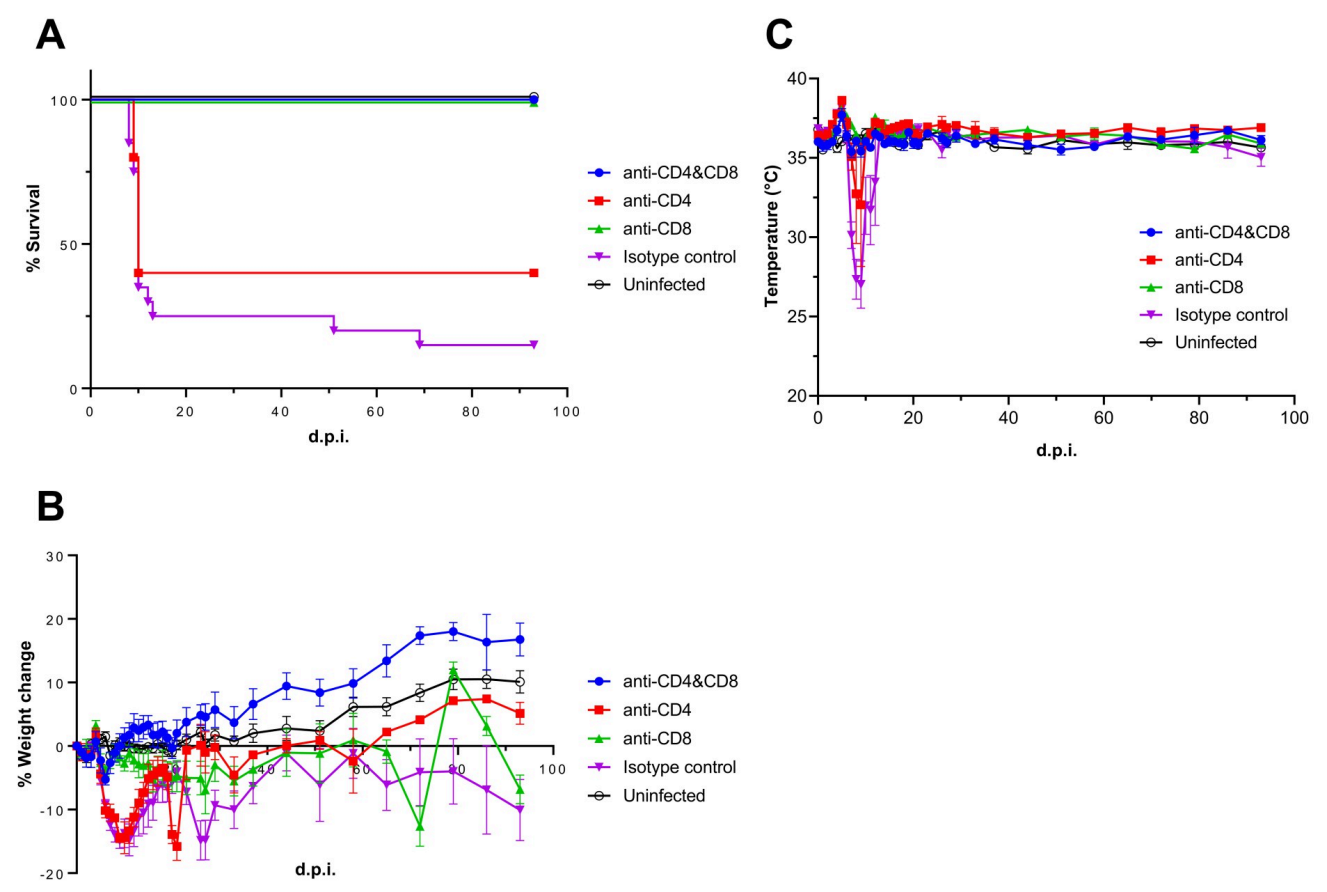
Pathogenicity of LASV in T-cell depleted Stat1-KO. Survival rate **(A)**, body weight change **(B)**, and body temperature **(C)** of T-cell-depleted Stat1-KO mice were plotted. Stat1-KO mice were injected with anti-CD4 and/or CD8 mAbs at 3 or 1 day prior to LASV infection in order to deplete CD4 and/or CD8 T-cells (n = 5). Mice in the isotype control group were injected with the isotype control mAb at 3 or 1 day prior to LASV infection (n = 20). Mice in the uninfected group were injected with the isotype control mAb at 3 or 1 day prior to PBS injection (n = 9).

**Fig 4:**
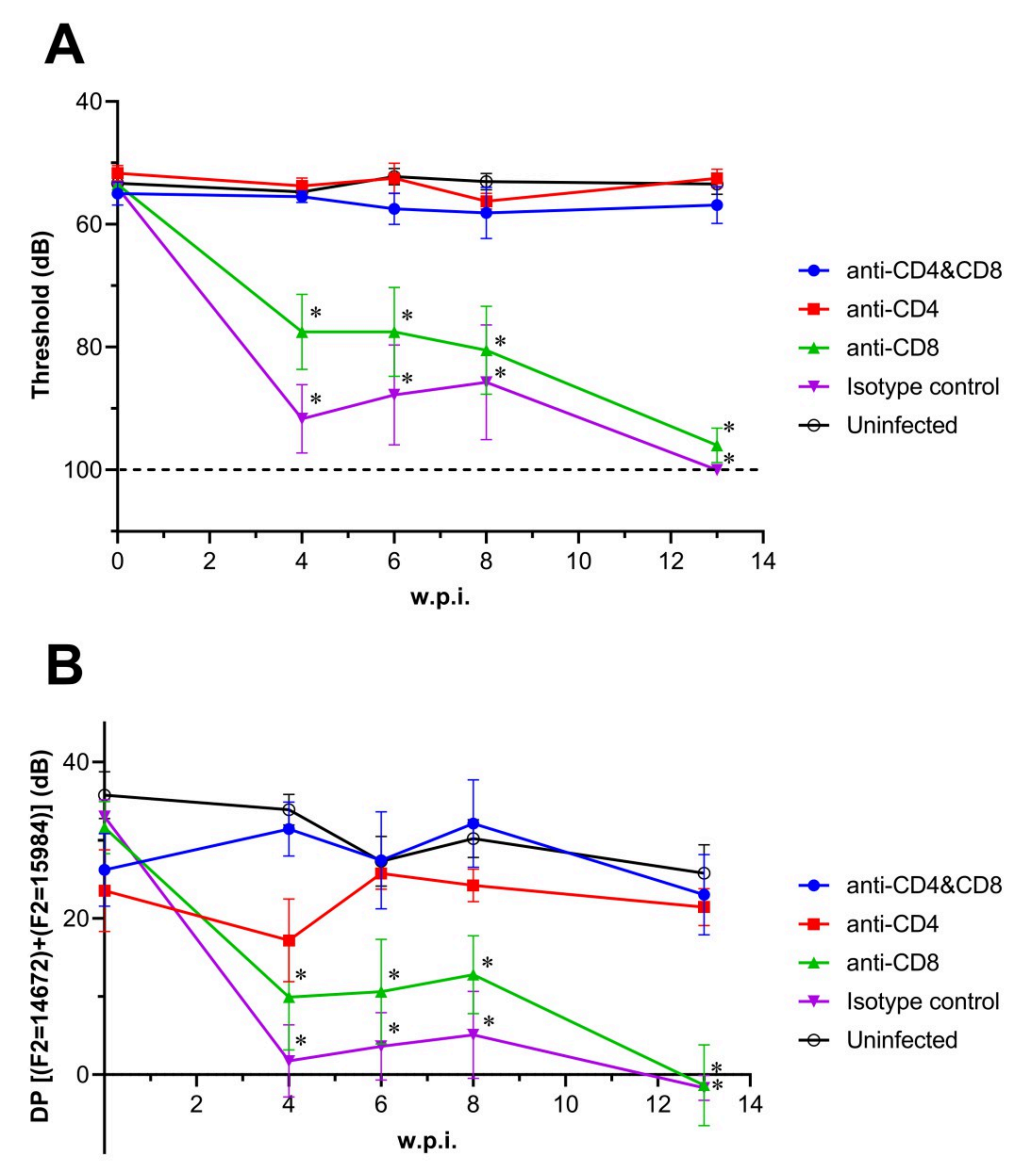
Hearing tests in T-cell-depleted mice infected with LASV. The results of ABR **(A)** or DPOAE **(B)** of LASV-infected Stat1-KO depleted with CD4 and/or CD8 T-cells were plotted. Data were shown using the mean and SEM. The broken line indicates the limit of detection in ABR (98< dB). Statistical differences at each time point compared to each uninfected group were calculated by 2-way ANOVA followed by Dunnett’s post hoc test, and shown in asterisks (p<0.05).

Our previous study indicated that T-cells infiltrated the inner ears of mice with hearing loss induced by LASV infection [14]. To assess the relationship between T-cells and LASV-induced hearing loss, we performed a T-cell depletion study using anti-CD4 and/or CD8 monoclonal antibody (mAb) injection. All mice in the CD4/CD8-double depleted or CD8-depleted group survived LASV infection while CD4 depleted or isotype control mAb injected mice showed 40% or 15% of survival, respectively (Fig 3A). All mice in the CD4-depleted or isotype control injected group showed weight loss and hypothermia starting at 6 d.p.i. with visible symptoms, such as scruffy coat, hunched back, and lethargy (Fig 3A and 3B). These acute symptoms disappeared at 14 d.p.i. in the surviving mice in accordance with previous data from Stat1-KO mice infected with LASV. Four out of 5 mice that survived LASV infection in the isotype control group developed neurological symptoms, such as imbalance and tremor starting at 26 d.p.i. while the survivors in the CD4-depleted group did not show any neurological symptoms in the later phase of LASV infection. On the other hand, all mice in the CD4/CD8-depleted group did not show any significant weight loss, body temperature change, or visible symptoms. Interestingly, the mice in CD8-depleted group did not show any weight loss or body temperature change in the acute phase of LASV infection with exception of a mild visible symptom, scruffy coat, at 6 and 7 d.p.i. (Fig 3B and 3C). However, all survivors showed weight loss starting at 3 w.p.i., and one of them developed neurological symptoms starting at 86 d.p.i. (Fig 3B). Both ABR and DPOAE results indicate that the survivors in CD8-depleted and isotype control infected group showed significant hearing loss starting around 4 w.p.i. which remained throughout the study (Fig 4A and 4B and S5 and S6 Tables).

### Antibody response and virus dissemination in T-cell-depleted Stat1-KO mice

**Fig 5:**
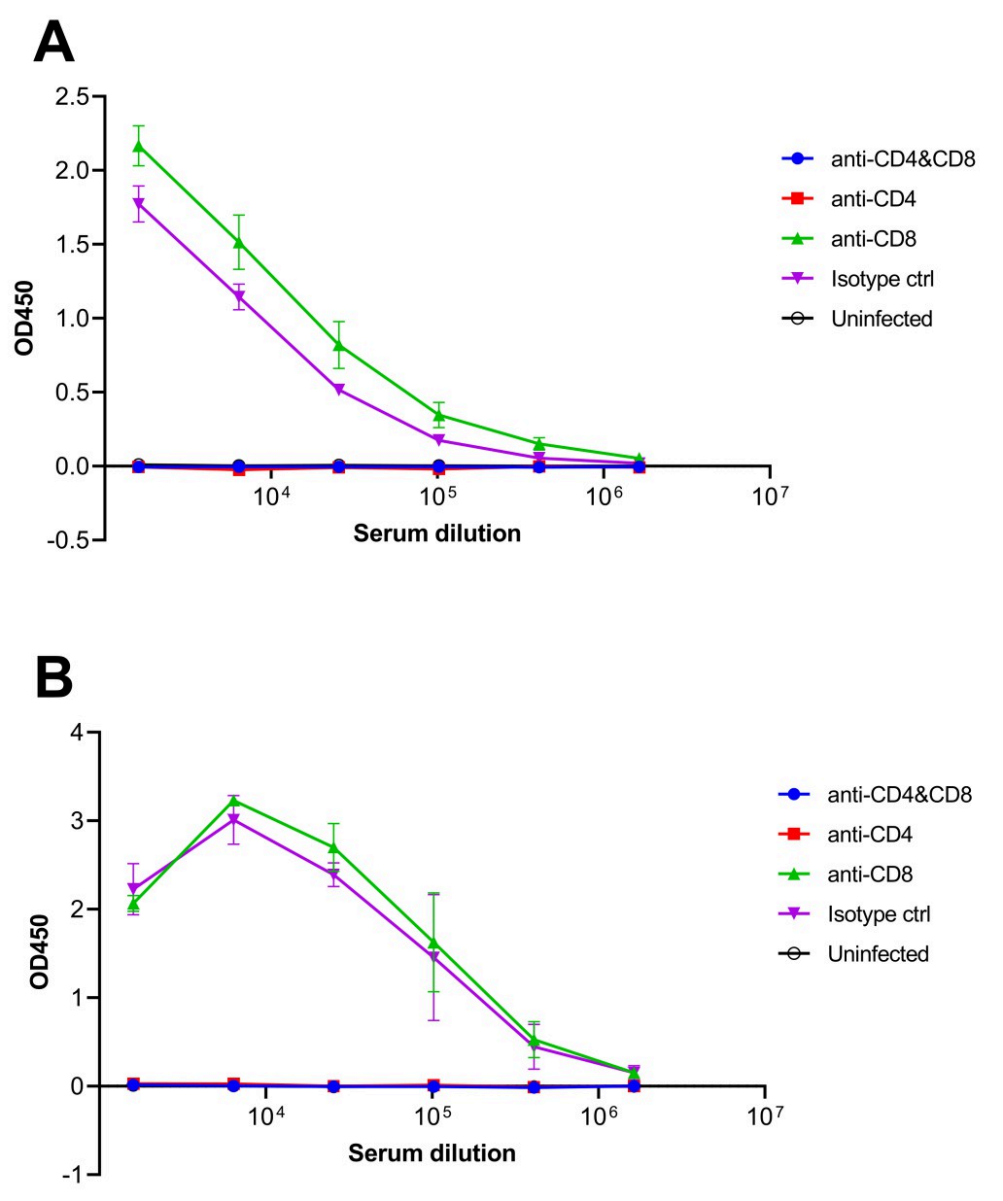
Antibody response against LASV in T-cell-depleted mice. Anti-LASV GPC **(A)** or NP **(B)** antibodies were detected by ELISA. Numbers of available serum samples are following; CD4/CD8-depleted group (n = 4), CD4-depleted group (n = 2), CD8-depleted group (n = 3), isotype control group (n = 2), and uninfected group (n = 5).

**Fig 6:**
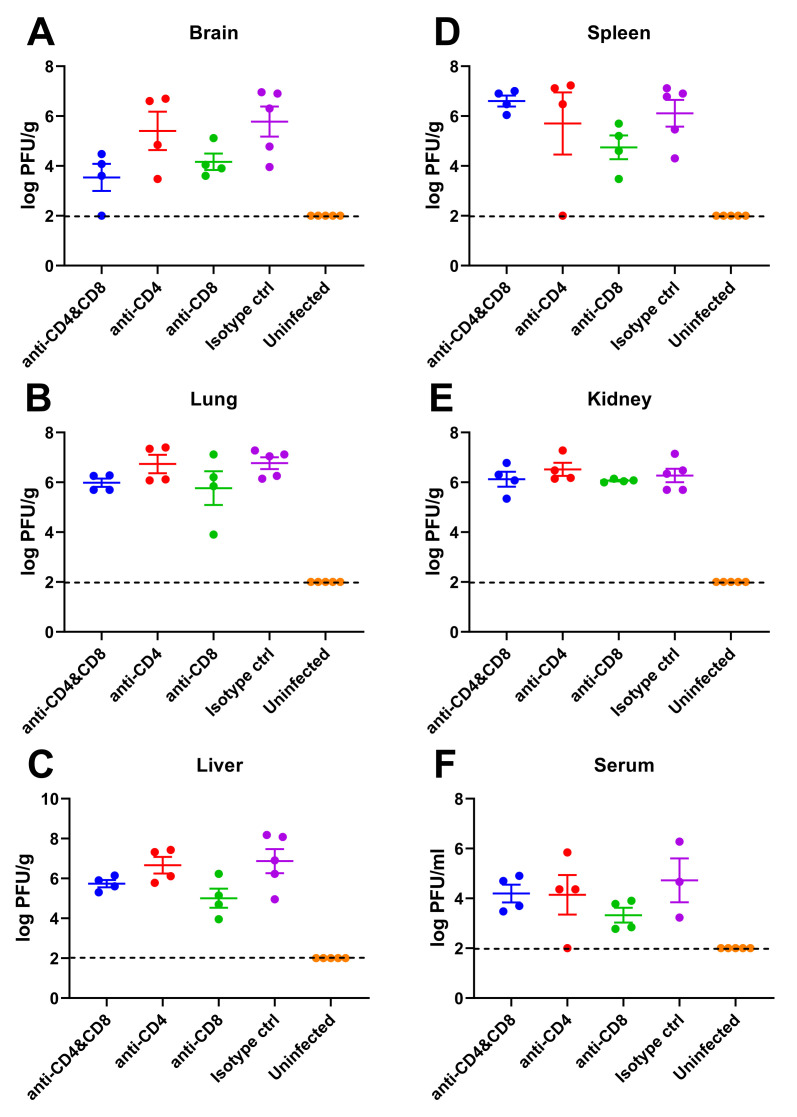
Virus dissemination in T-cell-depleted mice. LASV was detected in brain **(A)**, lung **(B)**, liver **(C)**, spleen **(D)**, kidney **(E)**, and serum **(F)** samples. The samples were collected at 98 d.p.i., excepting 2 mice in anti-CD4 group (8 and 9 d.p.i.) and 3 mice in isotype control group (8, 9, and 11 d.p.i.). Data from samples prior to 98 d.p.i. shown in open circles. The broken lines indicate the limit of detection (<2 logPFU/g for organ samples, and <2 logPFU/ml for serum samples).

Anti-LASV glycoprotein complex (GPC) or nucleoprotein (NP) antibodies in survivors were measured by Enzyme-linked immunosorbent assay (ELISA) (Fig 5). The mice in the CD8 depleted and isotype control groups showed an antibody response against both LASV GPC (Fig 5A) and NP (Fig 5B). On the other hand, the mice in the CD4/8-depleted and CD4-depleted groups did not show any antibody response against LASV GPC or NP, confirming that these antibodies are CD4 T-cell dependent. Virus titration results showed that LASV was present in all Stat1-KO mice even at the end of the study (7 w.p.i.) without any dramatic differences among groups, indicating that LASV caused prolonged infections in Stat1-KO mice (Fig 6).

### Histology of the inner ears in T-cell-depleted Stat1-KO infected with LASV

**Fig 7:**
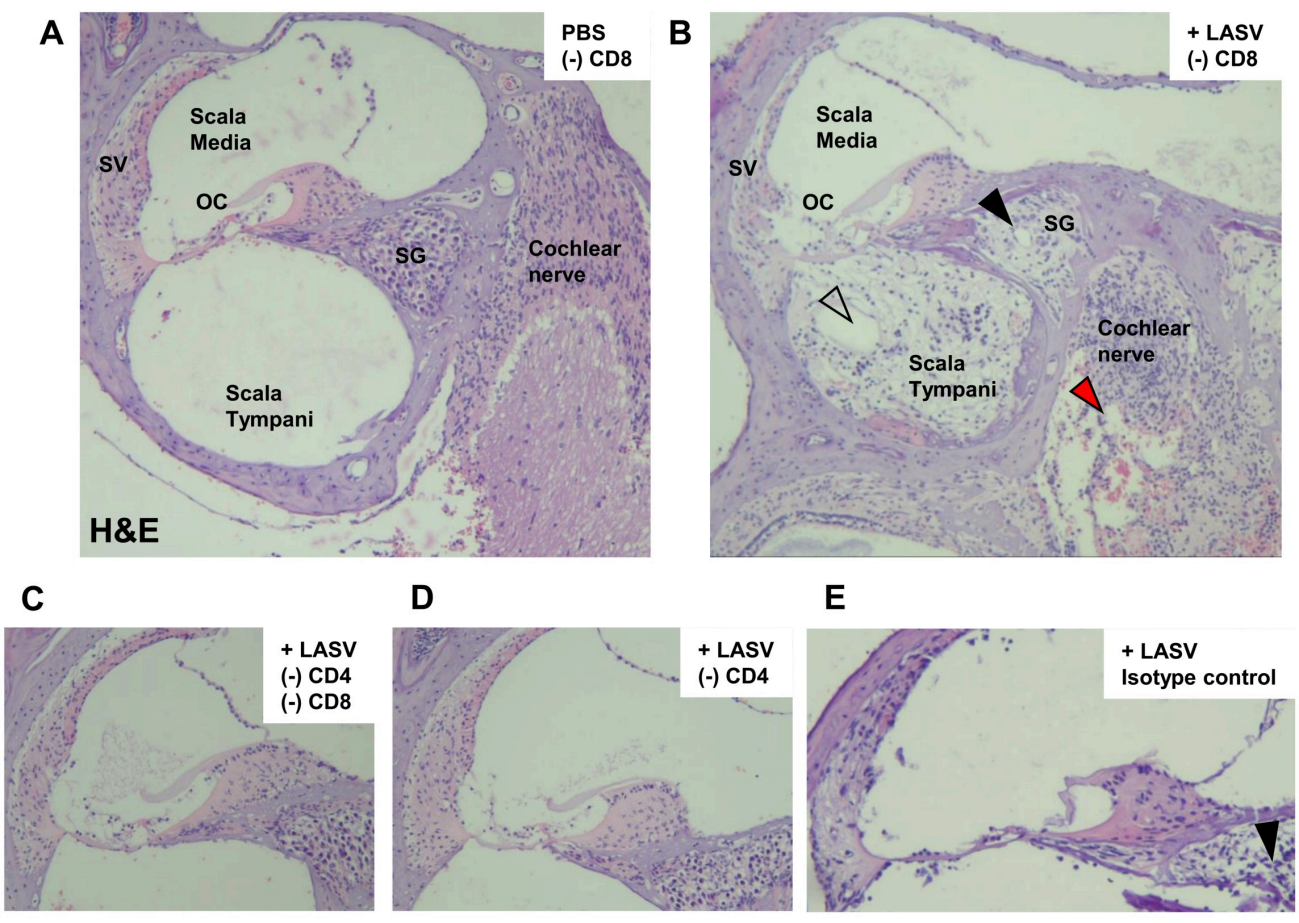
Damage on auditory neurons in LASV-infected mouse inner ear. Representative images of the cochlear ducts in the mouse from uninfected **(A)**, CD8-depleted **(B)**, CD4/8-depleted **(C)**, CD4-depleted **(D)**, isotype control group **(E)**. LASV infection induced significant damage to the inner ear in mice depleted of CD8 T-cells, whereas mice depleted with both CD4 and CD8 T-cells or depleted only with CD4 T-cells did not show any damage. Structural damage, hemorrhage and lymphocyte infiltration were observed in the spiral ganglion (black arrowhead), cochlear nerve (red arrowhead), and scala tympani (white arrowhead). Thinning of the stria vascularis (SV) was frequently observed associated with damage (B). Hemorrhage was observed in areas vascular-rich perineural areas. OC, organ of Corti; SG, spiral ganglion; SV, stria vascularis. 50x magnification. Samples were collected at time of euthanasia: (A) 98 d.p.i., (B) 98 d.p.i., (C) 98 d.p.i., (D) 98 d.p.i., (E) 93 d.p.i.

**Fig 8:**
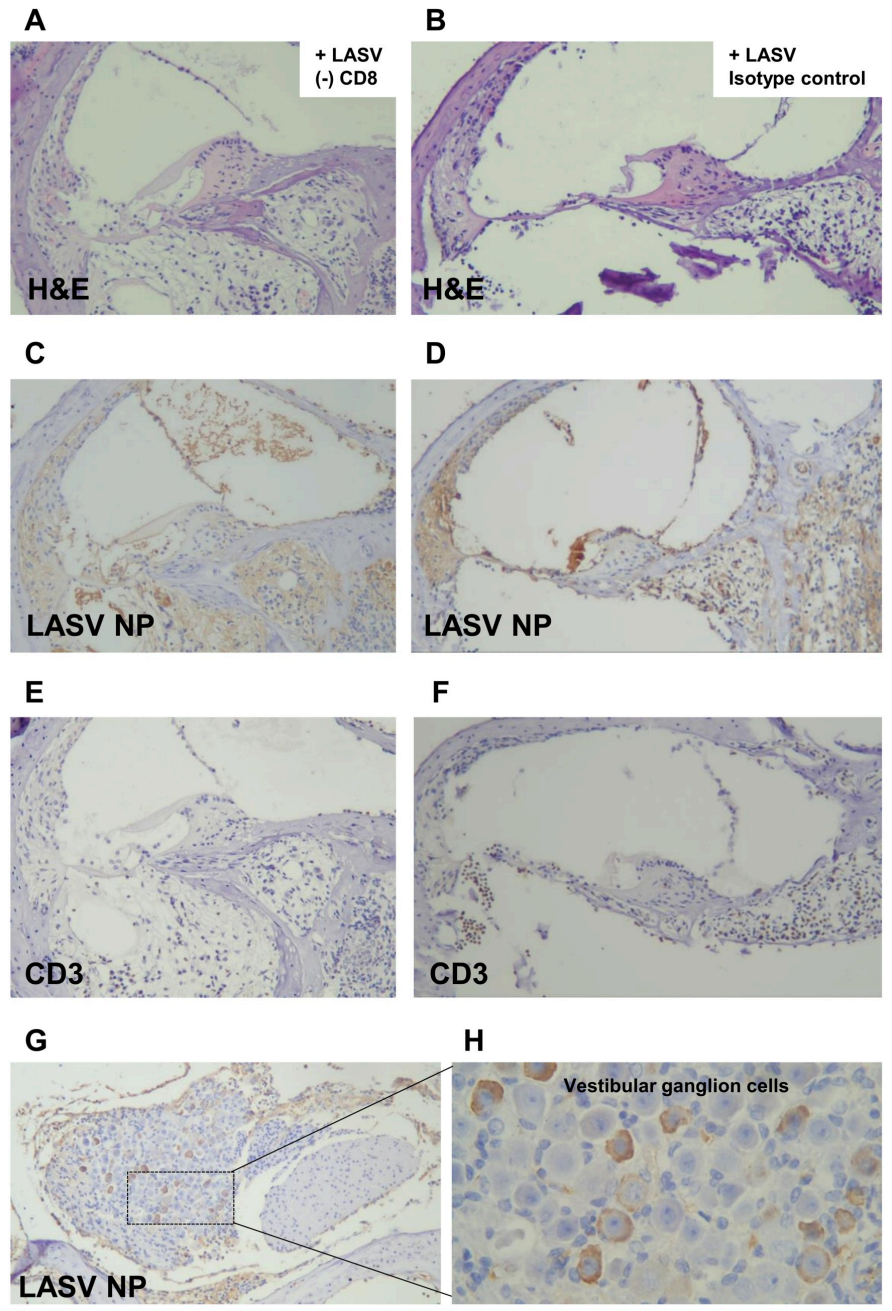
LASV antigen and CD3 positive lymphocytes in the inner ear. H&E staining **(A and B)** LASV NP antigen **(C and D)**, and CD3 positive lymphocytes **(E and F)** of scala vestibuli, scala tympani, and perineural areas. LASV NP antigen was also found in vestibular ganglion cells **(G and H)**. 100x **(A-F)**, 50x **(G)** and 400x **(H)** magnification. Samples were collected at time of euthanasia: (A) (C) (E) 98 d.p.i., (B) (D) (F) 93 d.p.i., (G) (H) 98 d.p.i.

Histological analyses of inner ears revealed that Stat1-KO mice infected with LASV showed significant damage, mainly to the neuronal structures within the cochlea (Fig 7E), consistent with our previous report [14]. In CD4/8-depleted or CD4-depleted mice infected with LASV, no damage to the cochlea was observed (Fig 7C and 7D). In contrast, in CD8-depleted mice infected with LASV, the cochlea showed significant damage comparable to that of the isotype control treated mice (Fig 7B and 7E). The damage was seen mainly in structures rich in neuronal cells such as the spiral ganglion, cochlear nerve, and in highly vascularized structures (S7 Table). Bulging of the Reissner’s membrane, suggestive of endolymphatic hydrops in the scala media, was observed in five out of eight (>60%) isotype control mice and in one out of four (25%) CD8-depleted mouse after LASV infection. The spiral ganglion cells were severely damaged, replaced with vacuoles and infiltrated with lymphocytes. Thinning of the vascular-rich stria vascularis was frequently observed in ears with severe damage. The LASV NP antigen and CD3 positive lymphocytes were detected in areas of destruction (Fig 8A–8F). LASV NP antigen was also observed in the vestibular ganglion cells with minimal destruction as compared to the spiral ganglion cells (Fig 8G and 8H).

## Discussion

In this study, we confirmed significant hearing loss in our mouse model of LF by ABR and DPOAE in the biosafety level 4 (BSL-4) laboratory. To our knowledge, this is the first report that conducted ABR and DPOAE in mice with the Risk Group-4 pathogen and in the BSL-4 facility. ABR and OAE analysis are non-invasive and quantitative clinical tests for human patients and can be adapted for high containment laboratory (BSL-4) usage in animal models of LF, as demonstrated in this paper. ABR detects neural activity in response to sound stimulus along the auditory pathway from the auditory nerve to the brainstem to the auditory cortex. Thus, the ABR can be used to localize the pathology along the auditory pathway in mild to moderate HL subjects by comparing the amplitude and latency of the response with normal subjects. On the other hand, OAE is associated with outer hair cell function within the cochlea. Unlike ABR, OAE reflects cochlear-specific function that is not influenced by any other pathology along the auditory pathway. Specifically, the DPOAE has advantages over other OAE modalities such that it can assess frequency-specific and tonotopic pathology within the cochlea. Hearing dysfunction was observed in both ABR and DPOAE in Stat1-KO mice infected with LASV but not WT mice. Our data revealed that hearing loss induced by LASV infection in Stat1-KO mice was detectable around the same time by ABR and DPOAE, indicating that LASV infection causes hearing loss through damage to the inner ears, as the main, early location of damages. Indeed, previous histopathology results [14] as well as the histopathology results from this study are consistent in that the neuronal structures such as the spiral ganglion cells and cochlear nerve were affected upon infection with LASV.

Several unique challenges exist surrounding administration of auditory function tests within the BSL-4 environment. In the highest biocontainment laboratory, ambient noise derived from the ventilation system, biosafety cabinet, or BSL-4 suit can directly interfere with the hearing tests. In addition, the noise in the housing facility is potentially greater than the normal 45–55 dB range [22], which may affect the auditory performance. Logistics in the BSL-4 lab are also challenging, which limits the total number of mice that can be tested per day. Based on our previous work with Stat1 deficient mice in which we reported no sex-dependent differences in auditory function [16], we designed our experiments to use female Stat1-KOmice for the ease of handling and housing. We chose to use the click sound to test the ABR because this stimulus resulted in the lowest threshold and consistent response in the Stat1 knockout mice. These modifications enabled us to complete the tests within 30 minutes per mouse. The ABR threshold at baseline in mice in the BSL-4 laboratory was indeed higher at around 55 dB HL compared to those in BSL-2 laboratory [16]. While this could result in a lack of early detection of mild to moderate hearing loss, the hearing loss measured in LASV survivors is adequately dramatic to overcome this limitation. For the DPOAE data analysis, we used the sum of the distortion products in the two highest F2 frequencies in our setup at 15kHz and 16kHz for data analysis, based on the observation that those two data points had robust response and less variance between male and female Stat1 knockout mice [16]. Even with many other potential obstacles, we detected a dramatic decrease in auditory function induced by LASV infection by both ABR and DPOAE.

Hearing loss in LASV-infected mice started around 3 w.p.i. and continued until the endpoint at 8 w.p.i. We speculate that at 3 w.p.i., the main etiology is continuation of the acute infection in the inner and middle ear, which then transitions into chronic infection and eventually an immune mediated damage to the neurons in the inner ear causing permanent hearing loss around 6 w.p.i. One of the limitations of our study is that we only have inner ear histology data for those mice that survived to the endpoint at 8 w.p.i. Future experiments with earlier time points can clarify our speculation on the transition from an infectious etiology to an immune etiology by direct comparison of auditory function with histology. Additionally, as hearing tests are unable to be performed during the acute phase of infection due to the severity of disease, we were unable to determine whether hearing loss occurs earlier than 3 w.p.i.

The Stat1-KO mice are known to develop middle ear infection spontaneously around 12–13 weeks of age resulting in unilateral or bilateral conductive hearing loss, which resolves in several weeks [23]. At the onset of hearing loss at 3 w.p.i., our Stat1-KO mice were around the susceptible age for developing middle ear infections. At the end point of our study at 8 w.p.i. the auditory function in our LASV-infected Stat1-KO mice were still in a trend of progressively declining, which suggests a different hearing loss etiology. In comparison, we observed only slight auditory function decline in mock infected Stat1-KO mice, which may reflect mild middle ear disease. Our histopathology studies showed significant damage to the neural and vascular structures in the cochlea of the LASV infected mice. There is a clear difference to the reported spontaneous middle ear infection in Stat1-KO mice with no apparent pathology in the cochlea [23].

There were similarities and differences in the inner ear pathology compared to mouse models of hearing loss induced by other viral infections. In Zika virus infected mice, at 9 days post-infection there was widespread damage to the cochlea and to the vestibular organs. The cochlear epithelium including hair cells, lateral wall, spiral limbus and spiral ganglion experienced more damage in the apex compared to the basal turn [24]. Tonotopical changes were seen in CMV infected mice, where the main damage in the vascular structure in the stria vascularis was in the mid-apical turn [25]. Our mice exhibited damage to the neuronal and vascular rich structures and in some cases with endolymphatic hydrops throughout all turns within the cochlea without clear tonotopic distribution. The limitation of our histological studies stems from the exhaustive regulative procedures required for tissue removal from the BSL-4 laboratory. Prolonged fixation with 10% formalin makes any downstream histological methods significantly challenging, in addition to already difficult manipulation of the mouse inner ear in general. In some of our samples, it was difficult to determine whether the damage was purely a result of the LASV infection, or artifact. Also, the later timepoint of our histological studies may be the reason for the lack of tonotopicity compared to other models. Future histology studies in earlier timepoints can clarify the difference.

Inoculation route in Zika virus studies was via footpad injection mimicking hematogenous spread whereas in CMV studies intraperitoneal [26,27], intracerebral [28–31], or placental [32] inoculations have been used. Regardless of the inoculation route, loss of spiral ganglion cells seems to be the most consistent histopathologic finding, followed by changes in stria vascularis. On the other hand, the organ of Corti and hair cells seem to be preserved in most cases even when hearing loss is present [27]. While inoculation route affects the prevalence of hearing loss in these mouse models [30], the overall histopathologic findings in the inner ear is very similar to our LF model mice. Perhaps these similarities suggest a common mechanism underlying viral infection induced hearing loss. Interestingly, in the CMV model, the degree of hearing loss and spiral ganglion cell loss were attenuated with corticosteroid administration suggesting inflammation as the etiology [27].

LASV induced HL has been compared to phenotypic similarities with sudden sensorineural hearing loss, a disease with unknown etiology that affect humans throughout the world [10,33]. Speculated etiologies include infectious, vascular, or autoimmune mechanisms [34]. There is an unusually high incidence of sensorineural hearing loss after LASV infection in adulthood [8] and there is no other known animal model of viral infection that can reproduce hearing loss with such high efficiency [14]. Thus, comparing inner ear histopathology in our mouse model with human inner ear histopathology may directly give clues on diagnosis and treatment of sudden hearing loss in humans. In temporal bone histopathology studies in patients with sudden hearing loss, the main finding was the loss of spiral ganglion cells mainly in the apical region of the cochlea. Degree of spiral ganglion cell loss was associated with the severity of hearing loss [35]. Similarly, our mouse model showed damage to the spiral ganglion cells. In future experiments, we hope to further use our model mice to test immune-modulating drugs to prevent or alleviate the hearing loss caused by LASV infection.

The previous study indicated T-cell involvement in LASV-induced SNHL [14]. We performed a T-cell depletion using anti-CD4 and/or CD8 mAb injection. No survivors in CD4/8- or CD4-depleted group showed SNHL indicating that CD4 T-cells play an important role. It is important to note that the high background noise within the BSL-4 facilites interferes with testing. Thus, small changes in ABR or DPOAE results indicating viral-mediated damage may have been missed during these studies. Future development of a surrogate model through which to study this hearing loss in low-containment facilities would eliminate this obstacle. CD4/8-or CD8-depleted mice developed no clinical symptoms in the acute phase of LASV infection indicating the importance of CD8 T-cells in the acute phase. The antibody response detected by ELISA demonstrated that CD8-depleted or isotype groups, which showed SNHL, had antibodies against LASV GPC or NP, however, all mice showed prolonged systemic infection. Taken together, we hypothesize the following: 1) Presence of CD8 T-cells is a key factor of LASV pathogenicity in the acute phase, 2) damage to the inner ears, and the resulting SNHL, may be due to a combination of viral dissemination and the immune responses contributed by CD4 T-cells. Previous study showed that IFNα/βγR-KO mice did not show hearing loss, in spite of LASV dissemination in inner ear, supporting that LASV-induced hearing loss are related to not only viral infection but also immune response [14]. The histopathology results also support this hypothesis. In addition, Cashman et al previously described the relationship between immune-mediated systemic vasculitis, in which LASV RNA was detected after viral clearance, and development of SNHL in LASV-infected *Cynomolgus macaques* [36]. Other work by Huynh et al in immunocompetent guinea pigs described the presence of pathological changes within the inner ear both in the presence and absence of viral antigen, supporting an immune-mediated mechanism as opposed to a direct viral-mediated mechanism [37].

An early and robust T-cell response, especially a CD4 T-cell response, is thought to be critical for protection and recovery against LF [38–42]. However, our results suggest that CD4 T-cells play an important role in LASV-induced SNHL. Stat1-KO mice have a partially immunodeficient phenotype, as Stat1 is a major player in the JAK/STAT pathway [43]. In fact, Stat1-KO mice have not eliminated LASV infection with or without T-cell depletion through 13 w.p.i. This prolonged LASV infection in Stat1-KO mice may be a key factor for a high rate of LASV-induced SNHL in this model. In other words, a partial immunodeficient condition allows for a prolonged LASV infection yet still induces an immune response, including humoral and cellar immunity against LASV, resulting in damage within the inner ear.

LASV infection is known to be immune suppressive, featuring a diminished T-cell response and lymphopenia [44,45]. Indeed, in human cases, it is hypothesized that LASV infection suppresses the immune response and causes an incomplete virus clearance, especially within the inner ears, allowing for SNHL to develop after the recovery of immune-related cells. This hypothesis leads to concerns about vaccination strategy against LF. In other words, vaccines against LF need to induce an immune response to prevent viral infection and dissemination effectively. Vaccines which can prevent symptoms, but not virus infection itself, potentially worsen the risk of SNHL as induced by LASV infection. Moreover, based on our data and current knowledge in the field of LF associated SNHL, we cannot exclude the possibility of autoimmunity induced by molecular mimicry upon infection. Therefore, it is very important to better characterize and understand this disease for future safer vaccine design and production.

The results obtained in this study suggest that a prolonged LASV infection within the inner ears in presence of detectable immune response(s), especially those involving CD4 T-cells, are responsible for LASV-induced SNHL. These findings have not only lead to new insights as to the mechanisms underlying LASV-induced SNHL, but should also pave way towards the development of countermeasures against it.

## Materials and methods

### Ethics statement

All work with infectious LASV was performed in biosafety level 4 (BSL-4) facilities in the Galveston National Laboratory (GNL) at the University of Texas Medical Branch (UTMB) in accordance with institutional guidelines. All animal studies were reviewed and approved by the Institutional Animal Care and Use Committee at UTMB and were carried out according to the National Institutes of Health guidelines.

### Cell and virus

Vero and HEK293T cells were maintained with in Dulbecco’s modified Eagle’s medium supplemented with 10% fetal bovine serum (FBS), 1% penicillin-streptomycin, and L-glutamine. LASV strain LF2350, which belongs to lineage IV, was isolated from non-fatal human LF case during a 2012 outbreak in Sierra Leone [14,46]. The virus was propagated in Vero cells, and virus-containing cell culture supernatant was stored in -80°C freezer until use.

### Virus titration

Virus titers were determined by plaque assay as described elsewhere [46]. Briefly, confluent monolayers of Vero cells in 12-well plates were inoculated with 100 μl of 10-fold diluted virus and incubated for 45 minutes at 37°C in CO_2_ incubator. After washing out the inoculum, each well was overlaid with minimum essential medium containing 2% FBS, 1% penicillin-streptomycin, and 0.6% tragacanth (Sigma). Following a 5- to 6-day incubation, cells were fixed with 10% Formalin and stained with Crystal Violet. Viral titers were represented as plaque forming unit (PFU).

### Virus challenge into Stat1-KO mice

Seven- to eight-week-old female Stat1-KO mice (129S6/SvEv-*Stat1*^*tm1Rds*^) or wild type (WT) 129S6 mice were purchased from Taconic Biosciences, Inc. All animals were housed in ABSL-2 and ABSL-4 facilities in the GNL at UTMB. Animal identification and measuring of body temperature were performed with subcutaneously implanted BMDS IPTT-300 transponders and a DAS-8007 transponder reader (Bio Medic Data Systems). Mice were intraperitoneally inoculated with diluted LASV strain LF2350 in 100 μl of PBS or 100 μl of PBS as an uninfected control. Mice were monitored illness with measuring body weight and temperature. Animals were humanely euthanized once they were unable to access their food or water, or lost more than 25% of their body weight.

### T-cell depletion

T-cell depletion was performed by intraperitoneal monoclonal antibody (mAb) injection. Mice were injected with 100 μg of InVivoPlus anti-mouse CD4 (GK1.5, BioXCell) [47] and/or anti-mouse CD8a mAb (53–6.7, BioXCell) [48] intraperitoneally at 3 and 1 day prior to LASV infection. InVivoPlus rat IgG2b isotype control and/or InVivoPlus rat IgG2a isotype control (BioXCell) were used as isotype controls. T-cell depleted mice were inoculated with 10^3^ PFU of LASV in 100 μl of PBS.

### Auditory tests

Mice were anesthetized with 100 mg/kg of ketamine and 10 mg/kg of xylazine via intraperitoneal administration. After becoming non-responsive to pinching of the tail or foot, the mouse was placed in a soundproof box for auditory tests. Auditory brainstem response (ABR) was measured by using the SmartEP platform (Intelligent Hearing System) as described previously [16]. Briefly, averages of 256 responses elicited by a click stimulus (broad band frequency, peak range 0.5 kHz– 16 kHz) delivered via ear insert in each ear were recorded in descending 5 dB steps. Threshold values were assigned at the lowest intensity at which an ABR was discerned. ABRs were measured in right and left ears individually in all mice used in this study before the experiments. If abnormal results were obtained from an ear at the baseline measurement, data from that ear was not used for the study. Distortion product otoacoustic emissions (DPOAE) was measured by using the Starkey DP2000 system (Starkey Laboratories) as described previously [16]. The distortion product (DP) at 2F_1_-F_2_ was measured at the high frequency setting with F_2_ values ranging between 8–16 kHz. The DP values from F_2_ = 14672 Hz and F_2_ = 15984 Hz were combined and used for analysis as DP values from the other frequencies were not clear due to high background noise in the ABSL-4 facilities. DPOAEs were measured in right and left ears indivisually in all mice used in this study before the start of the experiments. If abnormal results were obtained from an ear at the baseline measurement, data from that ear was not used for the study.

### Enzyme-linked immunosorbent assay (ELISA)

ELISA using LASV GPC or NP as antigens was conducted according to the previous report to detect antibody responses against LASV GPC or NP [49]. Briefly, plasmids expressing LASV GPC or NP were transfected into HEK293T cells and the cells were collected at 72 hour-post transfection. The collected cells were lysed with Mem-PER Plus Membrane Protein Extraction Kit (Thermo Scientific) or cell lysis buffer (50 mM Tris-HCl, pH 8.0, 300 mM NaCl, 0.5% Triton X-100) to extract LASV GPC or NP respectively. Empty vector transfected cells were used as negative control. Antibodies attached to the antigens were detected by rabbit anti-mouse IgG (light chain specific) mAb (HRP conjugate) (Cell Signaling) and visualized by adding 3,3’,5,5’-tetramethylbenzidine Liquid Substrate, Supersensitive, for ELISA (Sigma) and stopped with 1M phosphoric acid. The optical density at 450 nm (OD_450_) was measured and standardized with the OD_450_ of negative control antigens.

### Histological analysis

The mouse temporal bones were dissected from the head and kept in 10% formaldehyde for 7 days for fixation at the BSL-4 facility. The temporal bone was decalcified in 0.1 M EDTA in PBS for 7 to 14 days. The decalcified temporal bones were processed and embedded in paraffin, and the tissue was thin-sectioned at 5 μm onto slides. The slides were processed either for staining with hematoxylin and eosin (H&E) or for immunolabeling. After deparaffinization, the slides were processed for heat retrieval in pH 9.0 TE solution (Tris 10mM, EDTA 1mM) for 22 minutes in a rice cooker before immunolabeling. For immunohistochemistry, the slides were incubated in primary antibodies, Mouse anti-NP (Lassa Virus) monoclonal antibody (Cambridge Bio #01-04-0104) with 1:1000 dilution, or Polyclonal rabbit anti-Human CD3 antibody (Dako, #A0452) with 1:1000 dilution, overnight at 4°C, biotinylated secondary antibodies, Goat anti-mouse IgG Fc secondary antibody, Biotin (Invitrogen #SA5-10243) or SignalStain Boost IHC detection reagent (HRP, rabbit) (Cell Signaling Technology #8114) with 1:5000 dilution, for 30 minutes at room temperature, and then incubated in DAB substrate [ImmPACT DAB substrate, peroxidase HRP SK-4105 (Vector), or SignalStain DAB substrate kit #8059 (Cell Signaling Technology)] for 5 minutes, and counterstained with hematoxylin for visualization. Images were observed and captured using Leica DMLB microscope with Nikon Digital Sight 1000 digital camera and Nikon NIS Element F software.

### Statistical analysis

Statistical analyses were performed with GraphPad Prism Software (ver. 9.1.2). Statistical differences were calculated by 2-way ANOVA followed by Dunnett’s post hoc test for ABR and DPOAE results.

## Supporting information

S1 Table ABR results in Stat1-KO mice. (XLSX)

S2 Table DPOAE results in Stat1-KO mice. (XLSX)

S3 Table ABR results in WT mice. (XLSX)

S4 Table DPOAE results in WT mice. (XLSX)

S5 Table ABR results in T-cell depleted mice. (XLSX)

S6 Table DPOAE results in T-cell depleted mice. (XLSX)

S7 Table Histopathological change in inner ears. (XLSX)
